# Can facility delivery reduce the risk of intrapartum complications-related perinatal mortality? Findings from a cohort study

**DOI:** 10.7189/jogh.08.010408

**Published:** 2018-06

**Authors:** Rasheda Khanam, Abdullah H Baqui, Mamun Ibne Moin Syed, Meagan Harrison, Nazma Begum, Abdul Quaiyum, Samir K Saha, Saifuddin Ahmed, for the Projahnmo Study Group in Bangladesh

**Affiliations:** 1 International Center for Maternal and Newborn Health, Department of International Health, Johns Hopkins Bloomberg School of Public Health, Baltimore, Maryland, USA; 2 Johns Hopkins University-Bangladesh, Dhaka1213, Bangladesh; 3 International Centre for Diarrhoeal Disease Research (icddr,b), Bangladesh, Dhaka, Bangladesh; 4 Department of Microbiology, Dhaka Shishu Hospital, Dhaka, Bangladesh; 5 Department of Population, Family and reproductive Health, Johns Hopkins Bloomberg School of Public Health, Baltimore, Maryland, USA

## Abstract

**Background:**

Intrapartum complications increase the risk of perinatal deaths. However, population-based data from developing countries assessing the contribution of intrapartum complications to perinatal deaths is scarce.

**Methods:**

Using data from a cohort of pregnant women followed between 2011 and 2013 in Bangladesh, this study examined the rate and types of intrapartum complications, the association of intrapartum complications with perinatal mortality, and if facility delivery modified the risk of intrapartum-related perinatal deaths. Trained community health workers (CHWs) made two-monthly home visits to identify pregnant women, visited them twice during pregnancy and 10 times in the first two months postpartum. During prenatal visits, CHWs collected data on women’s prior obstetric history, socio-demographic status, and complications during pregnancy. They collected data on intrapartum complications, delivery care, and pregnancy outcome during the first postnatal visit within 7 days of delivery. We examined the association of intrapartum complications and facility delivery with perinatal mortality by estimating odds ratios (OR) and 95% confidence intervals (CI) adjusting for covariates using multivariable logistic regression analysis.

**Results:**

The overall facility delivery rate was low (3922/24 271; 16.2%). Any intrapartum complications among pregnant women were 20.9% (5,061/24,271) and perinatal mortality was 64.7 per 1000 birth. Compared to women who delivered at home, the risk of perinatal mortality was 2.4 times higher (OR = 2.40; 95% CI = 2.08-2.76) when delivered in a public health facility and 1.3 times higher (OR = 1.32, 95% CI = 1.06-1.64) when delivered in a private health facility. Compared to women who had no intrapartum complications and delivered at home, women with intrapartum complications who delivered at home had a substantially higher risk of perinatal mortality (OR = 3.45; 95% CI = 3.04-3.91). Compared to women with intrapartum complications who delivered at home, the risk of perinatal mortality among women with intrapartum complications was 43.0% lower for women who delivered in a public health facility (OR = 0.57; 95% CI = 0.42-0.78) and 58.0% lower when delivered in a private health facility (OR = 0.42; 95% CI = 0.28-0.63).

**Conclusions:**

Maternal health programs need to promote timely recognition of intrapartum complications and delivery in health facilities to improve perinatal outcomes, particularly in populations where overall facility delivery rates are low. The differential risk between public and private health facilities may be due to differences in quality of care. Efforts should be made to improve the quality of care in all health facilities.

The global burden of perinatal mortality is high and intrapartum complications are important contributors of perinatal deaths, particularly in low- and middle-income countries (LMIC) [1,2]. An estimated 15% of all pregnant women, experience acute severe intrapartum complications [3-5]. Intrapartum complications, such as prolonged labor, pre-eclampsia, maternal infections, and intrapartum hemorrhage are responsible for half of all maternal deaths, 23% of neonatal deaths, and 32% of stillbirths annually [6-8]. Considering both early neonatal deaths and intrapartum stillbirths, intrapartum complications cause about 2 million perinatal deaths each year [8,9].

A disproportionate share of the burden of perinatal mortality linked to intrapartum complications is seen in populations living in low and middle income countries (LMIC) where access to skilled care at birth is low; nearly all intrapartum related neonatal deaths and intrapartum stillbirths occur in LMIC [8,10]. South Asia and sub-Saharan Africa, settings with the lowest skilled birth attendance and highest intrapartum-related death rates account for 73% of intrapartum-related neonatal deaths globally [8,10]. The disparity is also apparent when comparing intrapartum stillbirths; stillbirth rates are 10 times higher in the poorest regions of the world compared to the richest [8]. It is in these countries with the highest burden of intrapartum complications-related deaths that the facility delivery rates are also the lowest, with only 44% and 48% of women delivering in facilities in South Asia and sub-Saharan Africa, respectively [11].

Strategies to reduce the burden of intrapartum complications in LMIC are known, and most of the perinatal deaths related to intrapartum complications can be prevented without high-cost interventions [12,13]. Perinatal survival has been linked to quality maternal and newborn care during pregnancy, especially during intrapartum and postpartum periods. The most effective interventions for preventing intrapartum related perinatal deaths are 1) antenatal care for early identification and management of pregnancy complications, 2) skilled birth attendance during delivery and 3) effective emergency obstetric care [12-15]. While strides have been made to increase prenatal care visits in LMIC settings, with two-thirds of women now accessing prenatal care services, only one-third actually access skilled care at birth, and even fewer receive immediate postnatal care [16].

One strategy to improve skilled birth attendance, access to effective emergency obstetric care and reduction of perinatal mortality is to increase the number of facility-based births. A systematic review by Lee et al. demonstrated that increasing skilled facility based births may reduce perinatal mortality by decreasing intrapartum-related deaths, and showed that high-intensity participatory community mobilization programs doubled the amount of institutional births, and prevented one-third of early neonatal deaths [17]. Simultaneously, improving health systems and facility readiness for prenatal, emergency intrapartum, and neonatal care coverage at both district and referral-level facilities can potentially reduce not only neonatal mortality and intrapartum stillbirths, but also maternal deaths [7]. These improvements include training of skilled birth attendants and improving facility capacities to provide life-saving interventions such as neonatal resuscitation and other emergency intrapartum care practices [16-18].

Additional evidence is needed, particularly from resource-constrained settings, to show whether facility based births can reduce the risk of intrapartum complications related perinatal mortality. Using data from a community based cohort study, the aim of this study is to examine the effect of intrapartum complications on perinatal mortality, and examine if delivering in a health facility can reduce the risk of perinatal mortality associated with intrapartum complications.

## METHODS

### Study design and implementation

This analysis used prospectively collected data from a community-based study conducted to determine the burden, etiology, and risk factors for community acquired neonatal infections. The study, Aetiology of Neonatal Infection in South Asia (ANISA), was a multi-centric study conducted in five sites of three countries of South Asia including a site in Sylhet district in Bangladesh. This paper used data from the Bangladesh site only and detailed of the study methods were published [19, 20].

Briefly, the study in Bangladesh was conducted in 14 unions (the lowest administrative unit with an average population of 28 500) of Kanaighat and Zakiganj sub-districts of Sylhet district in Bangladesh between June 2011 and December 2013. The households and the health facilities in the study area were mapped using Geographic Information System (GIS), and each household and household members have unique permanent identification numbers (PIDs) allowing longitudinal linkages. The study population was about 400 000 with an approximate annual birth cohort of 10 000. The perinatal mortality rate in Sylhet division was 63 per 1000 births [21].

The study data were collected by trained community health workers (CHWs), who were locally recruited women with at least a tenth grade education. In addition to study data collection, CHWs provided a basic package of maternal and newborn health (MNH) care to all women in the study area including counseling and education on preventive care, recognition of and care-seeking for maternal and newborn danger signs, and referral for emergency care during antepartum, intrapartum and postpartum periods [22]. CHWs routinely visited married women of reproductive age every two months at their homes and identified pregnant women based on reported last menstrual period (LMP). All consented pregnant women were enrolled in the study. The CHWs followed the pregnant women twice during the antepartum period and ten times during the postpartum period to provide the MNH care and to collect study related data.

### Data

During the first antepartum home visit, data were obtained on women’s household demographic and socioeconomic characteristics, their birth history including prior stillbirths, live births, and neonatal deaths, and data on their ability to make the decision to go to a health center alone for them and for their children. At the first postpartum home visit, within 7 days of delivery, data were collected on delivery, place of delivery, birth attendants, and history of self-reported intrapartum complications. All women were asked if they had experienced any of the following complications during the intrapartum period i) excessive bleeding during intrapartum period defined as bleeding that made the woman afraid of dying; ii) prolonged labour defined as labour lasting longer than 12 hours; iii) premature rupture of membranes (PROM) defined as rupture of the membrane more than one hour before start of labour; iv) abnormal presentation of baby; v) convulsion; and vi) retained placenta defined as failure to deliver the placenta for more than half an hour after the delivery of the baby. CHWs also obtained data on woman’s place of delivery ie, at home, in a public health or in a private health facility, and if the baby was born alive or dead. The average recall period for information for intrapartum complications, delivery care seeking and birth outcome was less than a week. The CHWs collected data on survival status of live born babies on day 28 after delivery and age at death for babies who died within 28 days of life.

### Measurements

**Figure 1:**
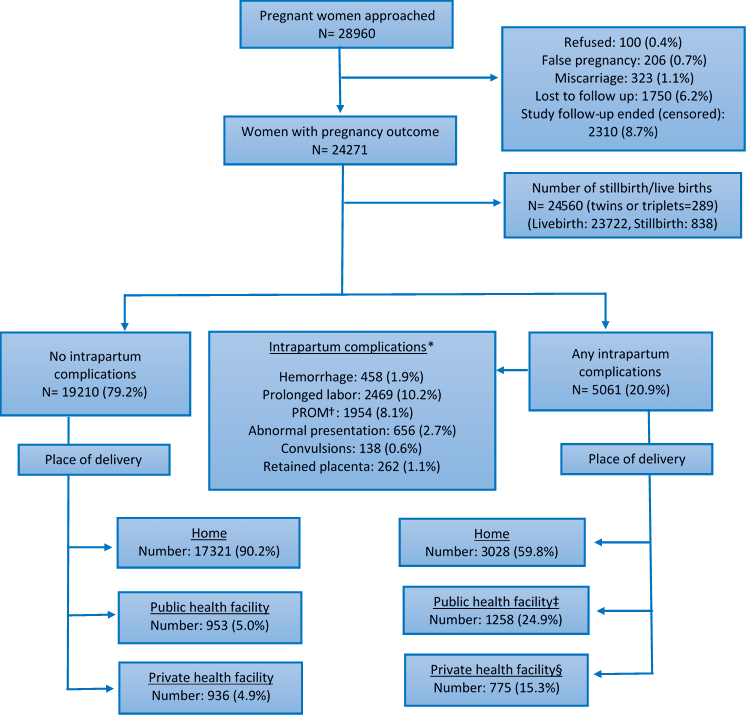
Intrapartum complications and place of delivery of a cohort of pregnant women in Bangladesh: 2011-2013. *Multiple responses; †PROM: premature rupture of membrane; ‡Public health facility included district hospital (DH), Upazila health complex (UHC), Upazila health and family welfare center (UHFWC); §Private health facility included private hospital, maternity center and clinic.

The main outcome variable is perinatal mortality defined as stillbirth (ie, birth of an infant that died in the womb after 28 weeks of gestation) or death of infant in the first 7 days of life (early neonatal mortality). We created a household wealth index as a measure of household economic status, using the Principal Component Analysis (PCA) method that used data on type of housing, source of drinking water, type of toilet, availability of electricity and household possessions (eg, availability of TV, radio, Cassette player, washing machine, water pump, electric fan, mobile phone, camera, clock, cooker, sewing machine, thresher, air condition, cart, car, scooter, bicycle, van, chair, bed, matt, sofa, table, cabinet, and domestic animal) a methodology generally used in the Demographic and Health Surveys [23]. The wealth index is a composite measure of a household's cumulative living and economic status. The wealth score places individual households on a continuous scale of relative wealth. We divided the households in to wealth quintiles. Thus, we have five groups ranging from the poorest to the wealthiest. Place of delivery was categorized into three groups: i) at home, ie, the delivery occurred in a place other than a health facility, ii) at a public health facility, ie, the delivery that occurred in a government owned and managed health facility, and iii) at a private health facility, ie, the delivery occurred in a privately owned and managed health facility. We created a composite binary exposure variable labelled as any intrapartum complications for women having any complication during intrapartum period (**Figure 1**) to increase statistical precision of the analysis.

### Statistical analyses

We examined the association between selected sociodemographic, economic, delivery characteristics, and intrapartum complications with perinatal mortality using Pearson χ^2^ test for independence. A p-value <0.05 was considered statistically significant. We examined unadjusted associations of selected sociodemographic and delivery characteristics, and each intrapartum complication with perinatal mortality. We then fitted two separate multivariable logistic regression models; the first model was to estimate odds ratios (OR) and 95% confidence intervals (CI) to determine the association of place of delivery with perinatal mortality regardless of intrapartum complications adjusting for covariates. In the second model, we examined if the association of intrapartum complications with perinatal mortality was modified by place of delivery using an interaction term. Data analysis was performed using STATA 14 (Stata Corporation 2015, College Station, TX, USA). We obtained approval from the Ethical Review Committee (ERC) of the International Centre for Diarrheal Disease Research, Bangladesh (icddr,b) and the Institutional Review Board (IRB) of the Johns Hopkins Bloomberg School of Public Health, USA to conduct the research.

## RESULTS

Of the 28 960 pregnant women who were approached to participate, 100 (0.3%) refused participation, 323 (1.1%) had a miscarriage, 1750 (6.2%) were lost to follow up, and 2310 (8.7%) women were not included because their pregnancy did not end during the study follow-up period (**Figure 1**). Pregnancy outcomes were recorded in 24 271 women resulting in 24 560 live or stillbirths including 289 twins and triplets. About 5061 (20.9%) of the pregnant women reported at least one intrapartum complication. The most common intrapartum complications reported were prolonged labor (10.2%) and PROM (8.1%) (Figure 1). Among women with any intrapartum complication, 3028 (59.8%) delivered at home, 1258 (24.9%) delivered at a public health facility, and the remaining 775 (15.3%) delivered at a private health facility (**Figure 1**). The mean (±standard deviation) age of enrolled women was 26.4 (±6.0) with a range of 13-54 years. About one quarter of the study women and more than one-third of their husbands had no formal education (**Table 1**).

**Table 1:** Association of perinatal mortality with selected sociodemographic, economic and delivery characteristics in Bangladesh

Characteristics	Total N = 24 560*	Survived during perinatal period	Died during perinatal period	UOR, 95% CI N = 24560	*P*-value
N = 22 972 (n, %)	N = 1588 (n, %)
**Age (years):**					
<20	2643	2415 (91.4%)	228 (8.6%)	Ref	<0.001
20-29	14374	13 526 (94.1%)	848 (5.9%)	0.66; 0.57-0.77	
≥30	7031	7030 (93.2%)	512 (6.8%)	0.77; 0.66-0.91	
**Religion:**					
Islam	23474	21 953 (93.5%)	1521 (6.5%)	Ref	0.680
Others	1086	1019 (93.8%)	67 (6.2%)	0.95; 0.74-1.22	
**Parity:**					
0	7735	6997 (90.1%)	738 (9.5%)	Ref	<0.001
1-2	9602	9172 (95.5%)	430 (4.5%)	0.44; 0.39-0.50	
3-4	4737	4483 (94.6%)	254 (5.4%)	0.54; 0.46-0.62	
**≥5**	2486	2320 (93.3%)	166 (6.7%)	0.68; 0.57-0.81	
**Family size:**					
1-4	7149	6638 (92.9%)	511 (7.2%)	Ref	<0.050
5-6	6782	6350 (93.6%)	432 (6.4%)	0.88; 0.77-1.01	
7-8	4755	4452 (93.6%)	303 (6.4%)	0.88; 0.76-1.02	
≥9	5874	5532 (94.2%)	342 (5.8%)	0.80; 0.70-0.92	
**Household wealth quintile:**					
Lowest quintile (poorest)	4960	4587 (92.5%)	373 (7.5%)	Ref	<0.001
Second lowest quintile	5090	4728 (92.9%)	362 (7.1%)	0.94; 0.81-1.09	
Middle quintile	4795	4478 (93.4%)	317 (6.6%)	0.87; 0.75-1.02	
Second highest quintile	4808	4509 (93.8%)	299 (6.2%)	0.82; 0.70-0.95	
Highest quintile (richest)	4907	4670 (95.2%)	237 (4.8%)	0.62; 0.53-0.74	
**Education:**					
No education	5785	5354 (92.6%)	431 (7.5%)	Ref	<0.001
1-5 years (primary)	8963	8379 (93.5%)	584 (6.5%)	0.87; 0.76-0.99	
≥6 years (secondary and above)	9812	9239 (94.2%)	573 (5.8%)	0.77; 0.68-0.88	
**Husband’s education:**					
No education	8520	7930 (93.1%)	590 (6.9%)	Ref	<0.01
1-5 years (primary)	8474	7902 (93.3%)	572 (6.8%)	0.97; 0.86-1.10	
≥6 years (secondary and above)	7566	7140 (94.4%)	426 (5.6%)	0.80; 0.70-0.91	
**Work for cash:**					
No	24 014	22 449 (93.5%)	1555 (6.5%)	Ref	0.680
Yes	546	513 (94.0%)	33 (6.0%)	0.93; 0.65-1.33	
**Women’s ability to make decision about child health care:**					
No	24 022	22 454 (93.5%)	1568 (6.5%)	Ref	<0.010
Yes	538	518 (96.3%)	20 (3.7%)	0.55; 0.35-0.87	
**Go to health center alone:**					
No	4144	3888 (93.8%)	256 (6.2%)	Ref	0.410
Yes	20 416	19 084 (93.5%)	1332 (6.5%)	1.06; 0.92-1.22	
**Past intrapartum history - any prior stillbirths or neonatal deaths:**					
No	22 225	20 839 (93.8%)	1386 (6.2%)	Ref	<0.001
Yes	2335	2133 (91.4%)	202 (8.7%)	1.42; 1.22-1.66	
**Trained ANC care:**					
No	9440	8806 (93.3%)	634 (6.7%)	Ref	0.210
Yes	15 120	14 166 (93.7%)	954 (6.3%)	0.94; 0.84-1.04	
**Place of delivery**					
Home	20 525	19 320 (94.1%)	1205 (5.9%)	Ref	<0.001
Public health facility	2268	1990 (87.7%)	278 (12.3%)	2.24; 1.95-2.57	
Private health facility/clinic	1767	1662 (94.1%)	105 (5.9%)	1.01; 0.82-1.24	
**Birth attendant:**					
Untrained†	1781	1670 (93.8%)	111 (6.2%)	0.66; 0.53-0.82	<0.001
TBA	18 297	17 231 (94.2%)	1066 (5.8%)	0.61; 0.54-0.69	
Skilled birth attendant‡	4482	4071 (90.8%)	411 (9.2%)	Ref	
**Distance from health facility (km):**					
0-5	4846	4542 (93.7%)	304 (6.3%)	Ref	<0.010
6-10	4846	4551 (93.9%)	295 (6.1%)	0.97; 0.82-1.14	
11-15	5535	5118 (92.5%)	417 (7.5%)	1.22; 1.04-1.42	
>15	9333	8761 (93.9%)	572 (6.1%)	0.97; 0.84-1.13	
**Hemorrhage:**					
No	24 092	22 568 (93.7%)	1524 (6.3%)	Ref	<0.001
Yes	468	404 (93.5%)	64 (13.7%)	2.35; 1.80-3.08	
**Prolonged labor:**					
No	22 056	20 821 (94.4%)	1235 (5.6%)	Ref	<0.001
Yes	2504	2151 (85.9%)	353 (14.1%)	2.77; 2.44-3.14	
**Premature rupture of membrane:**					
No	22 576	21 235 (94.1%)	1341 (5.9%)	Ref	<0.001
Yes	1984	1737 (87.6%)	247 (12.5%)	2.25; 1.95-2.60	
**Abnormal presentation:**					
No	23 874	22 438 (94.0%)	1436 (6.0%)	Ref	<0.001
Yes	686	534 (77.8%)	152 (22.1%)	4.45; 3.68-5.36	
**Convulsion:**					
No	24 421	22 854 (93.6%)	1567 (6.4%)	Ref	<0.001
Yes	139	118 (84.9%)	21 (15.1%)	2.59; 1.63-4.14	
**Retained placenta:**					
No	24 286	22 727 (93.6%)	1559 (6.4%)	Ref	<0.01
Yes	274	245 (89.4%)	29 (10.6%)	1.72; 1.17-2.54	
**Any complication:**					
No	19 404	18 496 (95.3%)	908 (4.7%)	Ref	<0.001
Yes	5156	4476 (86.8%)	680 (13.2%)	3.09; 2.79-3.44	

UOR – unadjusted odds ratio, CI – confidence interval, ANC – ante-natal care, TBA – traditional birth attendant

*Data are row percentages; comparing women with and without perinatal mortality.

†Untrained attendants included community health workers and relatives/neighbors.

‡Includes births assisted by skilled birth attendants (ie, doctors, nurses, midwives and paramedics).

In bivariate analysis, women’s age, parity, education, ability to make decisions on child care, previous history of stillbirth or neonatal death, place of delivery, type of birth attendants, any intrapartum complications, husbands’ education, family size, household wealth status, and distance to facility were significantly associated with perinatal mortality (**Table 1**). The risk of perinatal mortality was higher among women <20 years old (*P* < 0.001), primipara women (*P* < 0.001), and women from larger sized families (*P* < 0.05) (*P* < 0.001) (**Table 1**). Perinatal mortality was inversely-related to household wealth status; compared to women in the lowest wealth quintile, the risk was about 38% lower among women in the highest wealth quintile (**Table 1**). The risk of perinatal mortality was significantly higher among women who had a previous history of stillbirth or neonatal death (unadjusted odds ratio UOR = 1.42; 95% CI = 1.22-1.66) and who went to public health facilities for delivery (UOR 2.24; 95% CI = 1.95-2.57) (**Table 1**). All intrapartum complications that were examined in this study were associated with increased risks of perinatal mortality: hemorrhage (UOR = 2.35; 95% CI = 1.80-3.08), prolonged labor (UOR = 2.77 95% CI = 2.44-3.14), PROM (UOR = 2.25; 95% CI = 1.95-2.60), abnormal presentation (UOR = = 4.45; 95% CI 3.68-5.36), convulsion (UOR = 2.59; 95% CI = 1.63-4.14), and retained placenta (UOR = 1.72; 95% CI = 1.17-2.54) (Table 1). Women who had experienced any of the six above mentioned intrapartum complications were about three times more likely to experience perinatal mortality (UOR = 3.09; 95% CI = 2.79-3.44) compared to those who did not experience any intrapartum complications (**Table 1**).

In multivariable analysis, Model 1 shows that compared to women who delivered at home, women who delivered in a facility, either public or private, experienced an increased risk of perinatal mortality regardless of intrapartum complications. Delivering in a public health facility was associated with more than two times higher (OR = 2.40; 95% CI = 2.08-2.76) perinatal mortality and in a private health facility was associated with 1.3 times higher perinatal mortality (OR = 1.32, 95% CI = 1.06-1.64) compared to those who delivered at home after adjusting for covariates (**Table 2**). Model 2 shows that even among women with no intrapartum complications, delivering in a health facility, either public or private, was associated with higher risk of perinatal mortality compared to women who delivered at home (**Table 2**). Compared to women with no intrapartum complications and who delivered at home, the risk of perinatal mortality was more than three times higher (OR = 3.45; 95% CI = 3.04-3.91) than those who had intrapartum complications and delivered at home. Among women with intrapartum complications, delivering in a health facility was associated with lower risk of perinatal mortality compared to delivering at home. The risk was 43.0% lower for women delivering in a public health facility (OR = 0.57; 95% CI = 0.42–0.78) and 58.0% lower for women delivering in a private health facility (OR = 0.42; 95% CI = 0.28–0.63) (**Table 2**, Model 2).

**Table 2:** Association of factors with perinatal deaths for self-reported intrapartum complications and place of delivery

Risk factors	Model 1: SES and delivery characteristics (OR; 95% CI)	Model 2: SES and effect of place delivery (OR;95% CI)
**Age (years):**		
<20	Ref	Ref
20-29	0.64; 0.55-0.75	0.67; 0.57-0.78
≥30	0.70; 0.59-0.84	0.74; 0.62-0.88
**Family size:**		
1-4	Ref	Ref
5-6	0.88; 0.77-1.01	0.87; 0.75-0.99
7-8	0.90; 0.76-1.06	0.87; 0.74-1.01
≥9	0.86; 0.75-1.00	0.85; 0.73-0.98
**Household wealth quintile:**		
Lowest quintile (poorest)	Ref	Ref
Second lowest quintile	0.96; 0.82-1.12	0.95; 0.81-1.11
Middle quintile	0.90; 0.76-1.06	0.87; 0.73-1.03
Second highest quintile	0.86; 0.72-1.02	0.84; 0.70-1.00
Highest quintile (richest)	0.66; 0.54-0.81	0.65; 0.53-0.80
**Women’s education:**		
No education	Ref	Ref
1-5 years (primary)	0.90; 0.78-1.03	0.92; 0.79-1.06
≥6 years (secondary and above)	0.86; 0.73-1.01	0.86; 0.73-1.02
**Husband’s education**		
No education	Ref	Ref
1-5 years (primary)	1.04; 0.92-1.19	1.05; 0.92-1.19
≥6 years (secondary and above)	0.93; 0.80-1.08	0.95; 0.81-1.10
**Women’s ability to make decision about child health care:**		
No	Ref	Ref
Yes	0.58; 0.37-0.91	0.58; 0.36-0.91
**Previous history of stillbirths or neonatal deaths:**		
No	Ref	Ref
Yes	1.36; 1.16-1.60	1.35; 1.15-1.59
**Place of delivery:**		
Home	Ref	–
Public hospital	2.40; 2.08-2.76	–
Private hospital/clinic	1.32; 1.06-1.64	–
**Distance from health facility (km):**		
0-5	Ref	Ref
6-10	1.01; 0.84-1.18	1.05; 0.88-1.24
11-15	1.28; 1.10-1.50	1.31; 1.12-1.53
>15	1.07; 0.93-1.25	1.11; 0.95-1.28
Any intrapartum complication* place of delivery:		
No complication and home delivery	–	Ref
No complication but received delivery from public health facility	–	2.07; 1.62-2.65
No complication but received delivery from private health facility	–	1.46; 1.07-1.99
Had complication and home delivery	–	3.45; 3.04-3.91
Had complication and received delivery from public health facility	–	0.57; 0.42-0.78
Had complication and received delivery from private health facility	–	0.42; 0.28-0.63

SES –socio-economic status, OR – odds ratio, CI – confidence interval

*Denotes interaction between any intrapartum complication and place of delivery.

## DISCUSSION

In this population-based cohort of Bangladeshi pregnant women, the burden of self-reported intrapartum complication was high at 20.9%. The majority (59.8%) of the women with an intrapartum complication delivered at home, 24.9% sought delivery care form a public health facility, and 15.3% sought delivery care from a private health facility. Facility delivery was associated with increased risk of perinatal deaths regardless of intrapartum complications. The risk of perinatal deaths was 3.5 times higher among women who had intrapartum complications and delivered at home. Delivering in a health facility reduced the risk of intrapartum related perinatal mortality; risk was 43% lower when delivered in a public health facility and 58% lower when delivered in a private health facility.

In our population, the overall facility delivery rate was low, and those who delivered in a health facility experienced higher perinatal mortality compared to women who delivered at home. This finding was consistent with other studies conducted in Bangladesh, Nepal and Indonesia [24-26], where delivery by trained personnel were 34%, 8% and 33%, respectively. The higher risk of perinatal mortality among women who delivered in a health facility was presumably an effect of selectivity— these women might have had underlying medical conditions or complications that brought them to deliver in facilities [24,27]. A similar finding in Indonesia was explained by the fact that women usually chose facility delivery when they were sick or already experiencing complications, and often seek care too late to save lives [28]. Many maternal medical conditions that are potentially modifiable, such as maternal infections, non-communicable diseases, nutrition, and lifestyle factors have been showed to be associated with stillbirths [27] and early neonatal deaths. The high perinatal mortality is also associated with lack of timely care seeking and poor quality of delivery care in the health facility [24,29,30].

The rate of intrapartum complications we observed was similar to the rates observed in Bangladesh and elsewhere [31-33]. In a large population-based cohort study conducted between 2007 and 2011 in another area of rural Bangladesh, Sikder et al. observed that approximately 25% of the pregnant women reported having at least one intrapartum complication [32]. More than a quarter of women (28.5%) reported intrapartum complications in a cross-sectional study conducted in Ethiopia [33]. The higher perinatal mortality we observed among women with intrapartum complications is consistent with the findings from other countries [34-36]. Similar to finding from another Bangladesh study, we observed that the majority of the women with intrapartum complications did not seek facility based delivery care [37]. The higher risk of perinatal mortality among women with intrapartum complications who delivered at home compared to who delivered in health facilities is also consistent with a previous study [38].

The lower risk of perinatal mortality among women with intrapartum complications who sought care from health facilities compared to who delivered at home was differential by type of facility, although it was not statistically significantly different. The possible reasons for a lower risk of perinatal mortality in women who went to private health facilities compared to public health facilities may include socio-economic differences in the population, differences in the patient population in terms of severity of complications, and differences in service availability and quality. We do not have any data on severity of complications which is a potential limitation of the study. The Bangladesh Service Provision Assessment (SPA) survey conducted in 2014 suggests that availability of care for intrapartum emergencies were much higher in private than in public hospitals – availability of facilities for cesareans section were 33.4% in public compared to 75% in private health facilities and blood transfusion were 24.5% in public vs 56.8% in private health facilities [39].

According to Bangladesh Demographic and Health Survey (BDHS) 2014, only about one third (37%) of the Bangladeshi women delivered in a health facility [40]; facility delivery rate was lowest (22%) in Sylhet region where this study was conducted. Access to facility based delivery care is constrained by many factors including distance, lack of transport, cost, and generally poor quality of care in the facilities [41]. Several interventions on demand side financing, such as voucher schemes or conditional cash transfer, have shown potential to improving maternal health care utilization by increasing access and reducing inequity in low and middle income countries [42-44].

The findings that facility delivery is associated with perinatal mortality even when there was no intrapartum complications, and facility delivery in women with intrapartum complications reduce the risk of perinatal mortality emphasize the importance of increasing health facility delivery rates for all women, but particularly for women with intrapartum complications as recommended in the Lancet maternal heath series in 2006 and 2016 [45]. Although the health facility delivery rate in Bangladesh has increased from 12% in 2004 to 37% in 2014, the overall health facility delivery rate remained low. The rate was lowest in Sylhet division at 22.6% in 2014 (9.9% at public and 12.7% in private hospitals) compared to the national estimate [40]. A comprehensive approach to increase overall facility delivery rates including improvements in quality of care, early identification and management of intrapartum complications, and provision of emergency intrapartum and newborn care are essential to reduce perinatal mortality [2].

The study has several limitations. Self-reported intrapartum complications have limited validity, however, we used potentially serious and easily recognizable and reportable intrapartum complications by women themselves. We did not have quality of care data for this study; quality of care is associated with the risk of stillbirths and early neonatal deaths. The strengths of the study include a large sample size, population based prospective surveillance with independent identification of pregnancies, and short recall period that might have minimized recall errors of reported complications and adverse pregnancy outcomes. Since there is a potential for misclassification of stillbirth and early neonatal deaths, we decided to use a composite outcome, perinatal mortality.

The key programmatic finding from this study is that facility delivery should be promoted for all women, particularly for women with underlying medical conditions and intrapartum complications. Programs should promote early recognition of intrapartum complications and timely care seeking from an emergency intrapartum care facility. Availability of emergency care services is not universal and is more limited in public health facilities. According to recent health services assessments, only 10.2% of facilities are equipped to provide emergency intrapartum care services (ANC, normal and C-section) and 16.2% of facilities had blood transfusion services [39]. Although we could not study this, poor quality of care is also a major barrier to care seeking and a substantial impediment to improving maternal and perinatal health. To reduce perinatal mortality in settings such as ours, programs need to improve the availability of and access to quality maternal and newborn health services.
